# Wild and captive immature orangutans differ in their non-vocal communication with others, but not with their mothers

**DOI:** 10.1007/s00265-023-03426-3

**Published:** 2024-01-15

**Authors:** Marlen Fröhlich, Maria A. van Noordwijk, Tatang Mitra Setia, Carel P. van Schaik, Ulrich Knief

**Affiliations:** 1 Palaeoanthropology, Institute for Archaeological Sciences, Department of Geosciences, University of Tübingen, Tübingen, Germany; 2 Department of Evolutionary Anthropology, University of Zurich, Zurich, Switzerland; 3 Comparative Socioecology Research Group, Max Planck Institute of Animal Behavior, Konstanz, Germany; 4 Fakultas Biologi, Universitas Nasional, 12520 Jakarta Selatan, Indonesia; 5 Center for the Interdisciplinary Study of Language Evolution (ISLE), University of Zurich, Zurich, Switzerland; 6 Department of Evolutionary Biology and Environmental Studies, University of Zurich, Zurich, Switzerland; 7 Evolutionary Biology and Ecology, Faculty of Biology, University of Freiburg, Freiburg, Germany

**Keywords:** Behavioural plasticity, Communication, Gesture, Social interaction, *Pongo abelii*, *Pongo pygmaeus*

## Abstract

**Abstract:**

In many group-living species, individuals are required to flexibly modify their communicative behaviour in response to current social challenges. To unravel whether sociality and communication systems co-evolve, research efforts have often targeted the links between social organisation and communicative repertoires. However, it is still unclear which social or interactional factors directly predict communicative complexity. To address this issue, we studied wild and zoo-housed immature orangutans of two species to assess the impact of the socio-ecological setting on the production of non-vocal signal repertoires. Specifically, we compared repertoire size, dyadic repertoire similarity, and number of social goals (i.e. observer’s estimate of the signaller’s intended interaction outcome) for communicative interactions with mothers versus other conspecifics, controlling for critical individual and environmental factors. In this small sample of immature orangutans, wild-captive contrasts were statistically significant only for other-directed repertoires, but not for mother-directed repertoires, and not for the number of social goals that immatures communicated towards. While the repertoires of individuals living in the same research setting were more similar than those living in contrasting settings, this difference was most pronounced for other-directed repertoires of the less socially tolerant orangutan species. These results suggest that the boosted interactional opportunities in captivity rather than mere differences in environmental affordances or communicative needs drive the wild-captive contrast in orangutan communicative repertoires. Overall, this fine-grained analysis of repertoires further underscores that not only a species’ social organisation but also the targeted audience may have a profound impact on communicative behaviour.

**Significance statement:**

Navigating a dynamic social environment often requires flexible signal use. While it has repeatedly been shown that the social organisation and structure of species predict the complexity of their communication systems, the mechanisms underlying these relationships are largely unknown. Because targeted studies to assess this issue in great apes are difficult, we take an alternative approach here: we compare the same species living in the wild and in artificial habitats in captivity. This contrast allows a direct test of how repertoires respond to the relevant difference in socio-ecological conditions. Our results show that the diversity of interaction partners (i.e. social opportunities), but not the diversity of social goals (i.e. possible interaction outcomes) or the broader physical opportunities (i.e. safe ground use), predict the size and consistency of wild and captive signalling repertoires.

**Supplementary Information:**

The online version contains supplementary material available at 10.1007/s00265-023-03426-3.

## Introduction

Group-living animals often inhabit complicated social worlds. Navigating a dynamic social environment requires recognising group members, remembering past interactions with them and adaptively influencing their behaviour (Seyfarth and Cheney 2010). In essence, communicative signals serve to facilitate these social interactions by reducing uncertainty about the signaller’s intentions and likely behaviour (Cheney and Seyfarth 2018). In many social systems (e.g. those characterised by fission–fusion dynamics or a multi-level structure), unpredictability arises at multiple levels, including the identity of the interaction partner, the context of the social interaction, and the outcome (Freeberg et al. 2019; Peckre et al. 2019). These circumstances require individuals to flexibly modify their communication according to current social challenges by using social information, a phenomenon referred to as social competence (Taborsky and Oliveira 2012), reversible phenotypic plasticity (Dingemanse and Wolf 2013), or behavioural flexibility.

The link between social and communicative complexity in animal species (Freeberg et al. 2012) has gained increasing traction in recent years (e.g. Peckre et al. 2019; Rebout et al. 2020; Amici and Liebal 2022). The social complexity hypothesis for the evolution of communication posits that groups with complex sociality require more complex communication systems to regulate intraspecific interactions. Several studies have supported this hypothesis by revealing correlations between social and communicative variables. For the acoustic channel, there is extensive evidence from ground-dwelling sciurids (including marmots and prairie dogs) that diversity of social or demographic roles within groups predicts the size of the alarm call repertoire (Blumstein and Armitage 1997; Pollard and Blumstein 2012). For the visual channel, a recent study showed that higher levels of social tolerance in egalitarian societies, and thus higher uncertainty compared to living in a society with linear dominance hierarchies, are associated with more complex facial behaviour in macaque species (Rincon et al. 2023). For humans, a strong predisposition to social interaction lies at the core of their unique linguistic abilities (Levinson 2006; Tomasello 2008).

Nonetheless, the direction of causality and the mechanisms underlying these links remain unclear. While it is certainly undisputable that some aspects of animals’ social lives must be linked to their communication systems (after all, one fundamental function of communication is to navigate social interactions), we still do not understand which specific aspects of sociality or interaction predict a species’ communicative complexity—or vice versa (see also Peckre et al. 2019). We have learned that group size is not an adequate proxy for social complexity, as large aggregations of animals (e.g. the wildebeest herds in the Maasai Mara) do not always reveal differentiated inter-individual relationships (Bergman and Beehner 2015). Is it, instead, the number of social relationships and interaction partners that drive communication systems, their diversity or both? Even more importantly, how do *social relationships* versus *interaction partners* differ in their respective roles as social input?

In light of the staggering social diversity in primates, with their unique levels of social complexity among mammals (van Schaik 2016), behavioural studies within this taxon may help to shed light on these questions. We know that the socio-ecological environment primates grow up in has a profound impact on their communicative development (e.g. Snowdon and Hausberger 1997; Bard et al. 2014; Gillespie-Lynch et al. 2014; Fröhlich et al. 2017). Our understanding of developmental trajectories in great apes, our closest living relatives, has certainly improved over the past years, with studies scrutinizing early communication in captive (e.g. Tomasello et al. 1997a; Schneider et al. 2012) and now increasingly also in wild settings (e.g. Laporte and Zuberbühler 2011; Fröhlich et al. 2017; Bründl et al. 2022). Nonetheless, a direct comparison of immatures’ communication living in these different research settings (‘wild-captive contrasts’) is still lacking. The extent to which nonhuman primates in general and great apes in particular adjust their communicative behaviour in response to the immediate (‘behavioural flexibility’) or rather to the developmental environment (‘ontogenetic plasticity’) has critical implications for communicative innovativeness prior to the emergence of human language (Fröhlich et al. 2022).

In slowly-reproducing and rather solitary primate species like orangutans, mothers are presumed to have a particularly large influence on the development of their offspring’s competencies, including their communicative skills (Bard 1992; van Noordwijk and van Schaik 2005). However, temporary associations during feeding or travelling occur, particularly if food is abundant (MacKinnon 1974; Sugardjito et al. 1987), thus providing relatively rich opportunities for social interactions beyond the mother-infant unit (van Noordwijk et al. 2012; Schuppli et al. 2017; Fröhlich et al. 2020a). Given the widespread assumption that social interactions and opportunities are critical for the emergence of complex communicative exchanges (Snowdon and Hausberger 1997; Bard and Leavens 2014; Cheney and Seyfarth 2018), it is important to better understand how the time spent in temporary associations and interactions with social partners other than the mother affects the orangutans’ repertoire size, repertoire composition and functional use of signals.

Recently, we studied the extent of wild-captive contrasts in non-vocal communication repertoires in orangutans (Fröhlich et al. 2021a), the great ape genus for which the wild-captive contrast in socioecology is arguably greater than for any other hominid taxon. In captivity, orangutans do not compete for food resources, live at high densities and with less visual obstruction and are more ground-living than in the wild (Maple 1980; Liebal et al. 2006; Fröhlich and van Schaik 2020). This may favour the production of additional signals, so-called weak signal innovations (Ramsey et al. 2007; Lehner et al. 2010), which are completely or largely absent in the wild and thus cause differences in the communicative repertoires of individuals and groups (Fröhlich et al. 2021a). We adopted a 2 × 2 comparative approach, studying two species of orangutans that differ intrinsically in social tolerance, with Bornean orangutan being less socially tolerant and gregarious than Sumatran orangutans (van Schaik 1999; Mitra Setia et al. 2009), in both wild and captive settings. The study revealed pronounced wild-captive contrasts in non-vocal repertoire size on the individual and population level (with an 20% to 25% increase in the overall repertoire size of zoo-housed orangutans), as well as in the functional specificity of signal types. In addition, the similarity of repertoires was greater among any two individuals of the same (e.g. both wild) versus the opposing research setting (e.g. wild and captive). Hence, in orangutans facing conditions that allow for more sociability and ground-living, we may observe additional signals. Some of these may be latent, in that they are absent in the wild but reliably triggered in the greatly different captive conditions, others may be true, albeit weak, innovations. Likewise, another study found more elaborate communicative repair strategies in captivity than in the wild, but only in Bornean orangutans (Fröhlich and van Schaik 2022). Irrespective of species and setting effects, however, communicative acts may be adjusted to the interaction partner as a consequence of varying social tolerance and familiarity (see also Fröhlich et al. 2020b). Taken together, using an observational, comparative research paradigm we found that the ‘semi-solitary’ orangutans exhibit striking plasticity in their communicative behaviour.

With the ‘emergence’ of a more complex society, as we may see in certain social groups of captive orangutans, different group members have different roles to play, and the specification of appropriate respondents may require a considerable increase in the number of signal types used by a species (Marler 1967). This is independent of any increase in the number of response patterns (‘social goals’) that may occur. To date, it remains unclear whether the size and consistency of wild or captive signal repertoires is driven by a larger variety of interaction partners (i.e. social opportunities), the variety of social goals (i.e. possible interaction outcomes) that captive individuals need to communicate towards, or the broader physical opportunities (e.g. safe ground use) (Fröhlich et al. 2021a). Importantly, communicative repertoires may be differently expressed in interactions with mothers versus those with other conspecifics (e.g. peers or other adults), due to the high familiarity and social tolerance inherent to mother–offspring bonds. To tease apart the effects of these different sources of social input, a critical next step is to examine the effects of interaction partner and social goals on the production of communicative repertoires separately.

Here, we studied how social partners, age and observation effort influenced the non-vocal repertoire and its functional use in immature orangutans. Building on our previous work (e.g. Fröhlich et al. 2021a,b), we studied two species of orangutans that differ in social tolerance in both wild and captive settings (van Schaik 1999; Weingrill et al. 2011). To better understand the extent to which social opportunities matter for communicative diversity, we focussed on the differences between the non-vocal signal repertoire deployed towards mothers and the one deployed towards peers and other conspecifics (e.g. peers or unrelated adults), instead of using the entire repertoire expressed irrespective of interaction partner as commonly practised in primate communication studies. This decision allowed us to identify the specific influence that peers and other non-mother conspecifics have on communicative repertoires, letting us hopefully paint a fuller picture of the link between social and communicative complexity.

Specifically, we addressed three questions. First, we asked how infant age, orangutan species and research setting affected the *size* of both mother-directed and other-directed non-vocal signal repertoires (since vocalisations were very infrequent in this dataset on orangutans’ close-range interactions; see Fröhlich et al. 2021a). While it seems straightforward to expect an increase in the number of deployed signals throughout early life, it may also be possible that the maximum is reached before the end of the dependency period—especially in light of the semi-solitary lifestyle and long inter-birth intervals in orangutans. Thus, we expected age effects to vary depending on the interaction partner, along with effects of species and setting, but only for the repertoire size of other-directed signals.

Second, we investigated whether repertoire *composition* differed between research settings depending on the interaction partner, that is mothers versus others. To that end, we compared the repertoires of individuals living in the same (i.e. wild-wild, captive-captive) or contrasting research setting (wild-captive) using similarity indices (see also Fröhlich et al. 2021a, 2022). We predicted differences between within- and among-setting similarity only for the repertoire of other-directed, but not mother-directed signal repertoires, because mother–offspring interactions are expected to be similar in captive and wild settings.

Finally, we examined how infant age, research setting and species influenced the number of interaction outcomes immatures communicated towards and thus social goals obtained (Cartmill and Byrne 2010; Hobaiter and Byrne 2014), separately for mother- and other-directed signals. If interactions and repertoire sizes were largely driven by the range of social goals, one would predict that immatures have a larger variety of interaction outcomes with others, with age, and with a larger variety in captivity. Alternatively, if wild-captive contrasts in repertoires are caused by setting differences in socio-interactional opportunities rather than social goal variety, we would not expect effects of interaction partner, age and setting.

## Methods

### Study sites and data collection

This study is based on the same observational dataset on wild and captive populations of Bornean (*Pongo pygmaeus*) and Sumatran orangutans (*Pongo abelii*) as used in Fröhlich et al. (2021a), which was collected at two field sites and five captive facilities (zoos). We observed wild Bornean and Sumatran orangutans at the long-term research sites of Tuanan (Mawas Reserve, Central Kalimantan, Indonesia) and Suaq Balimbing (Gunung Leuser National Park, South Aceh, Indonesia), respectively. Both study sites consist mainly of peat swamp forest and show orangutan densities of 7 individuals per km^2^ at *Suaq* and 4 at *Tuanan* (Husson et al. 2009; Singleton et al. 2009). Captive Bornean orangutans were observed at the zoos of Cologne and Munster, and at Apenheul (Apeldoorn), while Sumatran orangutans were observed at the zoo of Zurich and at Hellabrunn (Munich). While captive Sumatran orangutans were housed in groups of nine individuals each, captive Bornean groups were generally smaller and sometimes included only a mother and her offspring (e.g. Apenheul). As opposed to previous studies that analysed the full sample of 71 individuals (i.e. including juveniles, adolescents and adults; Fröhlich et al. 2021a) or 26 mothers (Fröhlich et al. 2022), the present study focused only on dependent immatures: 13 immature Bornean (8 wild/5 captive) and 14 Sumatran orangutans (7 wild/7 captive) were included in the final sample (see electronic supplementary material, Table S1 for detailed information on subjects and sample sizes). It was not possible to record data blind because our study involved focal animals in the field.

### Data collection

Focal observations were conducted between November 2017 and October 2018 (Suaq Balimbing: November 2017–October 2018; Tuanan: January 2018–July 2018, European zoos: January 2018–June 2018). At the two field sites, these observations consisted of full (nest-to-nest) or partial follows (e.g. nest-to-lost or found-to-nest) of mother-infant units, whereas in zoos 6-h focal follows were conducted. Two different behavioural sampling methods were combined: First, all observed social interactions of the focal either as signaller or receiver with all partners, and among other conspecifics present (if the focal was engaged in a non-social activity while still in full sight) were recorded using a digital high-definition camera (Panasonic HC-VXF 999 or Canon Legria HF M41) with an external directional microphone (Sennheiser MKE600 or ME66/K6). In captive settings with glass barriers, we also used a Zoom H1 Handy recorder that was placed in background areas of the enclosure whenever possible. Second, using instantaneous scan sampling at ten-minute intervals, we recorded complementary data on the activity of the focal individual, the distance and identity of all association partners, and in case of social interactions the interaction partner as well as several other parameters. During ca. 1600 h of focal observations, we video-recorded more than 6300 social interactions which were subsequently screened for good-enough quality to ensure video coding.

### Video coding procedure

This study is based on 8219 (wild setting: 3512, captive setting: 4707) high-quality recordings of orangutan immatures’ communicative acts directed either at mothers (*N* = 3446; wild: 2521, captive: 925) or other conspecifics (*N* = 4773; wild: 991, captive: 3782) which were previously coded using the program BORIS v. 7.0.4. (Friard and Gamba 2016). The focus was mainly on gestures, defined as socially directed, mechanically ineffective movements of the extremities, head or body or body postures (e.g. Pika 2008; Fröhlich et al. 2017), thus including both manual and bodily communicative acts, but also facial expressions (see Table S2 for full repertoire of immatures). All individual gestures and facial expressions were defined and aligned based on previous studies on orangutan intentional communication in captive (Jantschke 1972; Zucker et al. 1978; Liebal et al. 2006; Cartmill and Byrne 2010) and wild settings (MacKinnon 1974; Rijksen 1978; Fröhlich et al. 2019; Knox et al. 2019). We also coded the ‘presumed social goal’ as the human observer’s estimate of the signaller’s intent based on behavioural context (including co-locomote, share food/object, groom, play/affiliate, move away, sexual contact and stop action; Cartmill and Byrne 2010; Fröhlich et al. 2021a) as well as the interaction outcome (i.e. whether the signaller ceased communication and if it represented the signaller’s plausible social goal; Hobaiter and Byrne 2014).

To ensure inter-coder reliability, we evaluated the coding performance of all observers using the Cohen’s Kappa coefficient (Bakeman and Quera 2011). After an initial training period of 2 to 4 weeks, and afterwards in regular intervals (once a month), consistency of coding performance between at least two observers was evaluated with different sets of video recordings (10 to 20 clips each) using the Cohen’s Kappa coefficient to ensure inter-coder reliability (Bakeman and Quera 2011). Trained coders proceeded with video coding only if at least a ‘good’ level (κ = 0.7) of agreement was found for signal type, presumed goal, and interaction outcome. All trained observers were ignorant of the study’s aims. Final (average) levels of agreement were κ = 0.87 for signal type, κ = 0.86 for presumed goal and κ = 0.73 for interaction outcome. A detailed overview of individual observers, the study groups coded and final inter-observer reliability scores for our key variables is provided in the supplementary material (Table S4).

### Statistical analyses

In this study, we only included immature subjects that contributed to more than 30 interactions with either mothers or others, and only considered signal types that were used at least twice by a particular subject, to obtain more conservative measures of the size and composition of individuals’ customarily used repertoires. Importantly, this means that the sample size per interaction partner category was not necessarily the same for both settings (one of the captive immatures was an orphan, and several of the wild infants did not yield sufficient data for the analysis of other-directed repertoires). Out of the full sample of 27 immatures, 25 individuals (8/4 wild/captive Borneans, 7/6 wild/captive Sumatrans) were available for the analyses of mother-directed repertoires, and 21 individuals (4/5 wild/captive Borneans, 5/7 wild/captive Sumatrans) for the analyses of other-directed repertoires (see electronic supplementary material, Table S1).

To examine sources of variation in the size of immatures’ mother-directed and other-directed repertoires (i.e. number of non-vocal signal types used at least twice; question 1), we fitted two separate generalized linear mixed models (GLMMs; Baayen 2008) with a Poisson error structure and repertoire sizes as our response variables. Because same-aged (‘peers’) and older interaction partners may affect repertoires differently, we also fitted models separately for peer- (same-aged) and older-directed repertoires (for definitions see Fröhlich et al. 2020a), even though sample sizes were technically too small for inferential analyses (see Results). In all models, we included age (years; both as simple and squared term), research setting (2 levels: captive, wild) and orangutan species (2 levels: Bornean, Sumatran) as our key predictor variables. We included sex (2 levels: female, male) and the observation effort (range = 32–776 signal instances; first log- and then z-transformed to approximate linearity) as additional fixed effects (i.e. control predictors) in the model. To control for repeated measurements within the same sampling unit, group ID (i.e. zoo or field site) was treated as a random intercept effect. The same procedure of model specification was followed for the analysis on the sources of variation in the number of social goals (question 3).

All models were implemented in R (version 4.0.3; R Core Team 2020) using the function *glmer* of the package lme4 (Bates et al. 2014). To control for collinearity, we determined the Variance Inflation Factors (VIF; Quinn and Keough 2002; Field 2005) from a model including only the fixed main effects using the function *vif* of the R package car (Fox and Weisberg 2011). This revealed no collinearity issues (maximum VIF = 3.1), which also shows that observation effort did not simply correlate with research setting. We checked for overdispersion using a self-written function by Roger Mundry (see also Fröhlich et al. 2017), revealing no issue (all dispersion parameters < 1). Model stability tests (see also Nieuwenhuis et al. 2012; Fröhlich et al. 2021b) revealed that effects of all predictor variables were at least moderately stable (that is, the minima and maxima of the case-wise deletion of levels of random effects did not deviate substantially from the original estimates, and there was no shift from the positive to negative range or vice versa). To test whether the combined effect of our key test predictors were statistically significant (Forstmeier and Schielzeth 2011; Mundry 2014), we compared the full models with the respective null models comprising only the control predictors as well as all random effects using a likelihood ratio test (Dobson 2002). Tests of the individual fixed effects were derived using likelihood ratio tests (R function *drop1* with parameter ‘test’ set to ‘Chisq’).

To assess between-individual variation in mother-directed versus other-directed non-vocal signal repertoires and to compare repertoire similarity within and between research settings (question 2), we calculated Dice coefficients *D*_*C*_ (Dice 1945) for each pairing of individuals as *D*_*C*_ = (2 × *C*_*AB*_) / (*R*_*A*_ + *R*_*B*_), where *C*_*AB*_ is the number of signal types observed in both individuals (A and B), and *R*_*A*_ and *R*_*B*_ being the number of signals in the repertoire of individuals A and B. The values range between 0 (no signals in common) and 1 (identical signal repertoires) (see Halina et al. (2013) and Fröhlich et al. (2021a) for similar analyses). Because this results in a matrix of pairwise comparisons between individuals, in which each individual contributes to multiple comparisons, data points are non-independent row- and column-wise, and require a permutation-based construction of the null distribution of Dice coefficients. Thus, we conducted matrix permutations (*N* = 1000 permutations) in R (R Core Team 2020) to assess whether (i) immatures of the same settings (wild-wild and captive-captive pairings) share more types of mother- and other-directed signals than immatures living in contrasting settings (wild-captive pairings), and (ii) immatures living in captive settings have more dissimilar mother- and other-directed repertoires than individuals in wild settings. Thus, for this analysis the zoos had to be pooled for each species. We predicted these differences because we included more different captive than wild groups/matrilines per species (two or three zoos versus one field site), but also because social life in captivity is more terrestrial (i.e. freeing hands for communication) and more diverse in terms of partner variety (see also Fröhlich et al. 2021a). In the matrix permutation test, we used the differences between contrasting groups to calculate *P* values. Because the distribution of these differences does not necessarily need to be symmetric around zero and because these differences could either be negative or positive, we used significance thresholds of *P* ≥ 0.975 and *P* ≤ 0.025, rather than the more conventional *P* ≤ 0.05 *P* value cut-off.

## Results

### Production of mother- and other-directed signals across settings

Among our dataset of 8219 communicative acts, we identified a total of 35 signal types that were directed at mothers, while all 41 previously identified non-vocal signal types (Fröhlich et al. 2021a) were directed at other conspecifics (see Table 1 for numbers broken down in relation to species and settings, and Table S2 for detailed repertoires). Thus, there were six signal types (*flap lip*, *head*-*stand*, *hit ground/object*, *shake object*, *spin*, *spit*, see Table S2) in the total repertoire that were never directed at mothers across both species and research settings.

**Table 1:** Overall number of non-vocal signal types in relation to research setting, orangutan species and interaction partner

	Captive		Wild		All
	Bornean	Sumatran	Bornean	Sumatran	
Mothers	31	28	25	27	35
Others	37	34	15	31	41

### Repertoire size of mother- and other-directed signals

**Fig. 1:**
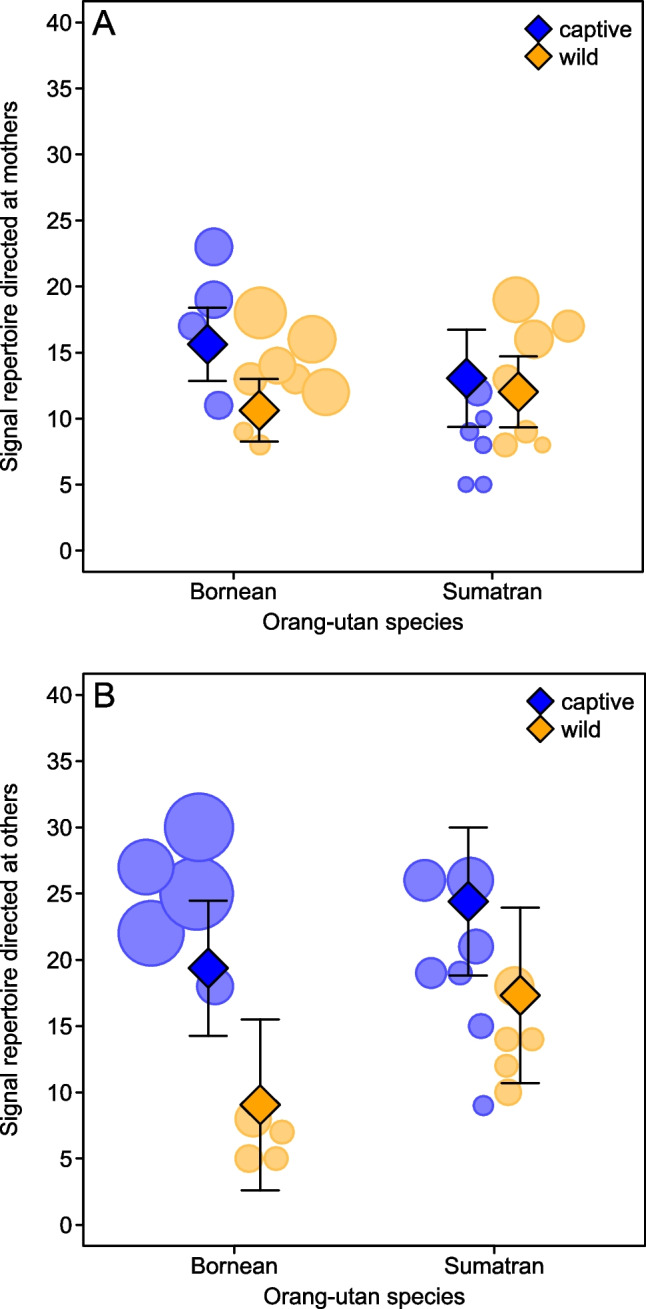
Number of observed signal types per individual directed at **A** mothers and **B** others as a function of research setting and orangutan species. Circles represent different individuals with area corresponding to sample size; diamonds depict model estimates with 95% confidence intervals (all other variables centred to a mean of zero)

First, we tested whether age, species and research setting affected the number of different signal types (i.e. repertoire size) produced in interactions with both mothers and others. Likelihood ratio tests (LRTs) revealed contrasting findings for mother- and other-directed repertoires: While the full model did not explain behavioural variation better than the null model for the number of mother-directed signal types (LRT: *χ*^2^_4_ = 4.74, *P* = 0.315, *N* = 25; Fig. 1A), we found a significant effect of key test predictors for the number of other-directed signal types (LRT other-directed: *χ*^2^_4_ = 13.052, *P* = 0.011, *N* = 21; Fig. 1B). More specifically, other-directed repertoires were smaller in wild compared to captive settings (estimate ± SE =  − 0.42 ± 0.191, *χ*^2^_1_ = 4.837, *P* = 0.028), larger in Sumatrans compared to Borneans (estimate ± SE = 0.518 ± 0.171, *χ*^2^_1_ = 8.124, *P* = 0.004), and when more signal instances were recorded (estimate ± SE = 0.349 ± 0.108, *χ*^2^_1_ = 7.411, *P* = 0.006; see also Table S3).

To understand whether a certain type of partner (i.e. same-aged versus older) drove this result, we conducted two follow-up analyses, teasing apart signal repertoires directed at same-aged partners (‘peers’) from those directed at older (non-mother) partners (see also Fröhlich et al. 2020a). Obviously, given our threshold of 30 signal cases for inclusion, this resulted in considerably smaller sample sizes (peer-directed repertoires: 7 wild versus 8 captive individuals; older-directed repertoires: 4 wild versus 12 captive individuals) available for analyses. For peer-directed repertoires, the difference between wild and captive settings was still significant (estimate ± SE = − 0.421 ± 0.213, *χ*^2^_1_ = 4.003, *P* = 0.045), but not in a model for older-directed repertoires (estimate ± SE = − 0.132 ± 0.258, *χ*^2^_1_ = 0.262, *P* = 0.609), probably due to the large wild-captive discrepancy in sample size. Although these findings should be viewed with caution, this still shows that opportunities for interactions with older non-mother social partners were considerably rarer in the wild than in captivity. Together, these results demonstrate that wild-captive contrasts in repertoire sizes mainly arise from interactions with same-aged or older individuals beyond the mother–offspring bond.

### Within- and between-setting repertoire similarity for mother- and other-directed signals

**Fig. 2:**
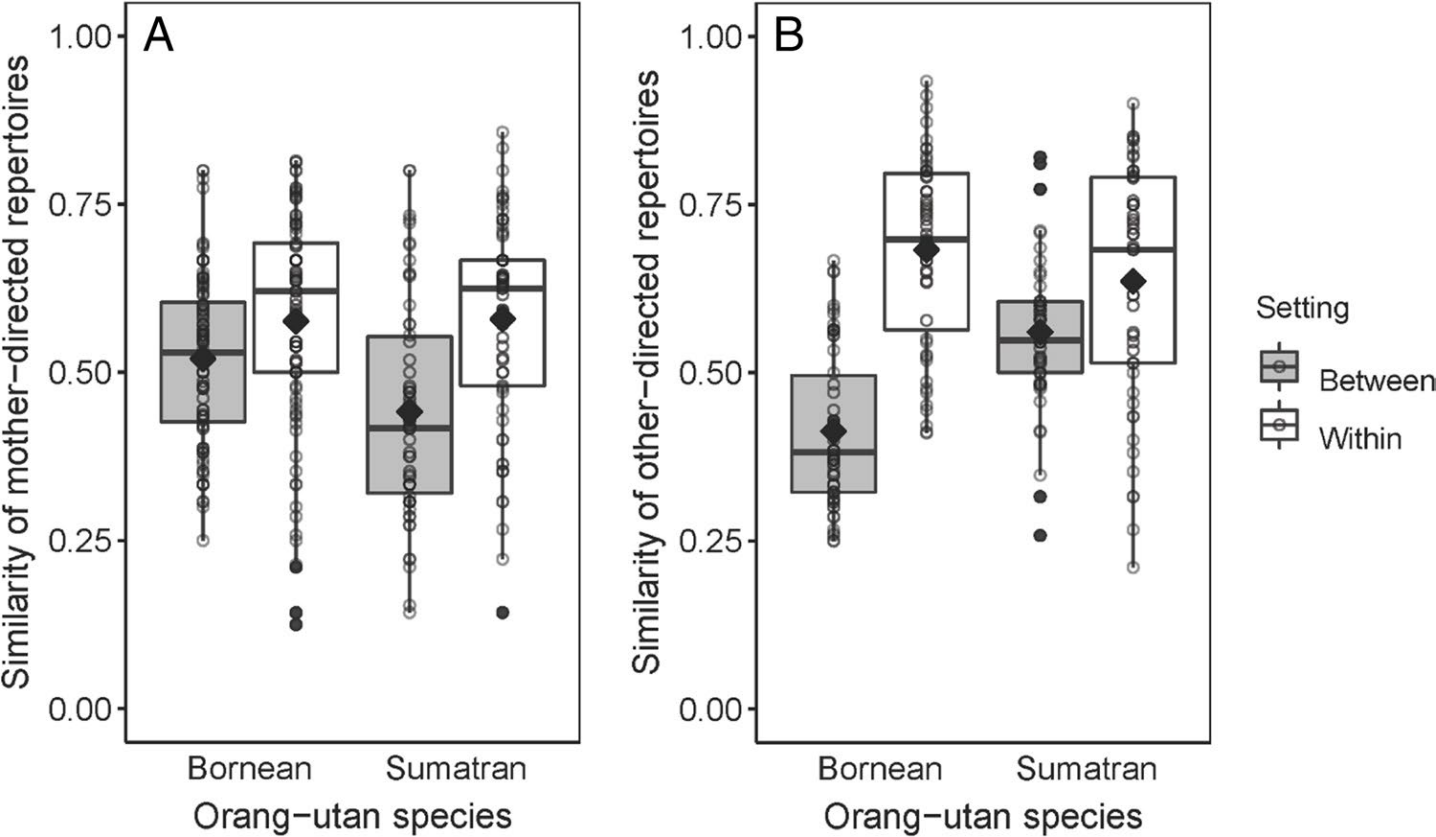
Similarity of non-vocal signal repertoires directed at **A** mothers and **B** others between pairs of immatures living in different (‘between’) and the same (‘within’) research settings, separately for each orangutan species. Indicated are dyadic Dice coefficients (circles), population means (filled diamonds), medians (horizontal lines), quartiles (boxes), percentiles (2.5% and 97.5%, vertical lines) and outliers (filled dots). Individuals contributed to multiple data points

Second, we examined to what extent the composition of mother- and other-directed repertoires expressed by immature orangutans overlapped between and within research settings (captivity or wild). More specifically, we tested whether Dice coefficients compared within settings (i.e. degree of concordance in repertoires between two individuals living in the same research setting) differed systematically from Dice coefficients compared between settings. We conducted permutation tests separately for both orangutan species. Regardless of species, both mother- and other-directed repertoires differed at least moderately between individuals, with particularly low similarity of other-directed repertoires among immatures that live in contrasting (‘between’) settings (Fig. 2; Table 2). As previously shown for the infant-directed repertoires of orangutan mothers (Fröhlich et al. 2022), communicative repertoires of individuals living in the same setting (within-setting similarity) were more similar than those of individuals living in contrasting settings (between-setting similarity; Borneans mother-directed: *P* < 0.001, Sumatrans mother-directed: *P* < 0.001, Borneans other-directed: *P* < 0.001, Sumatrans other-directed: *P* < 0.001). Moreover, visual inspection of the plot revealed that the difference between within- and between-setting overlaps was much more pronounced for other-directed repertoires, as predicted.

**Table 2:** Mean Dice coefficients indicating similarity of non-vocal repertoires directed at mothers and others between pairs of immatures living in different (‘between’) and the same (‘within’) research settings, separately for each orangutan species

	Mother-directed		Other-directed	
	Bornean	Sumatran	Bornean	Sumatran
D_c_ within settings	0.69	0.56	0.83	0.72
D_c_ between settings	0.58	0.40	0.36	0.58
D_c_ within settings (only captive)	0.68	0.37	0.83	0.71
D_c_ within settings (only wild)	0.69	0.69	0.82	0.73

In the previous analysis, the within-setting measures from captive and wild individuals were pooled. In a second analysis, we separated within-wild from within-captive individuals, because these comparisons might also differ from each other in meaningful ways, even though such an analysis would be less robust given the sample size limitations. This additional analysis showed that degrees of repertoire overlap within captivity versus within wild did not significantly differ, except for the mother-directed repertoires in Sumatran immatures (see Table 2; Borneans, mother-directed; matrix permutation test: *P* = 0.46; Sumatrans, mother-directed: *P* = 0.006; Borneans, other-directed: *P* = 0.47; Sumatrans, other-directed: *P* = 0.42). These results should be viewed with caution, however, since sample sizes within specific settings were obviously small (e.g. only four zoo-housed Bornean immatures contributed to the within-captivity scores, in contrast to eight Bornean immatures in the wild).

Consistent with previous work including interactions beyond the mother–offspring dyad (Fröhlich et al. 2021a), our results suggest that communicative repertoires used in the wild and in captivity systematically differ in composition. This strengthens the finding that immatures in captivity use not only a greater number, but also a different variety of other-directed signal types than do immatures in the wild.

### Number of interaction outcomes involving others versus mothers

Finally, we tested whether age, species and research setting affect the number of different outcomes observed in immatures’ interactions with both mothers and others. For both datasets, the full model did not explain variation better than the null model (LRT mother-directed acts: *χ*^2^_4_ = 0.202, *P* = 0.995, *N* = 25; LRT other-directed acts: *χ*^2^_1_ = 6.728, *P* = 0.151, *N* = 21). These results suggest that the variety of social goals immatures communicated towards does not systematically differ between ages, orangutan species and research settings.

## Discussion

Although the support for a co-evolution of sociality and communicative repertoires is growing, the direction of causality and the mechanisms underlying these links remain obscure (Freeberg et al. 2012; Pollard and Blumstein 2012; Peckre et al. 2019). Not only the social organisation and therefore the variation in the audience may influence communicative behaviour, but also the individual characteristics of a targeted recipient (Fröhlich et al. 2016; Graham et al. 2020). We addressed this issue by investigating whether the impact of the socio-ecological environment on the size and composition of non-vocal signal repertoires differs depending on immature orangutans’ interaction partners, that is, their mothers versus other conspecifics. Our results indicated that the size of immatures’ repertoire directed at non-mother conspecifics such as peers was higher in captivity than in the wild and among Sumatrans than among Borneans, while no such differences were found for the repertoire of mother-directed signals. Given the relatively small sample size of 27 individuals for this analysis, these results should be treated with caution (see below). Nevertheless, they provide tentative evidence that the diversity of interaction partners provided by captivity (especially non-immature but also same-aged interaction partners) affects the communicative repertoire in the *Pongo* genus: individuals in both the wild and captivity and regardless of species, all have a mother, whereas multiple different partners are available in captivity. As expected, the difference between wild and captive settings in other-directed repertoires was largest for Borneans, the less sociable orangutan species. At the same time, our analyses revealed that Sumatran immatures had significantly larger other-directed repertoires, irrespective of the research setting and despite the small number of individuals. This was expected because wild Sumatran (Suaq) orangutans are exposed to a social setting much more similar to captivity than those wild Borneans in Tuanan, with the latter having far fewer opportunities to interact. As mentioned above, such a wild-captive contrast was not found for mother-directed repertoires and not for the diversity of social goals that individuals communicated towards. However, we again need to highlight that an effect of the small sample size cannot be excluded.

Another, subtler difference became apparent when analysing the *composition* of non-vocal signal repertoires directed at mothers versus others. In line with previous work including all age classes (Fröhlich et al. 2021a), permutation tests showed that individuals living in the same research setting had more similar repertoires than those in contrasting settings, irrespective of interaction partner and orangutan species. However, this difference was markedly less pronounced for mother-directed repertoires than for other-directed repertoires in the Bornean species. On the one hand, these results are consistent with the view that new affordances of captive settings enabled orangutans to better exploit their (communicative) motion spectrum, resulting in novel communicative movements that may independently and predictably be produced in several captive colonies and species (Lehner et al. 2010; Fröhlich et al. 2021a). On the other hand, this lends further, albeit preliminary, support to the notion that interaction partners more than physical-environmental affordances drive wild-captive contrasts in signal use. All six non-vocal signal types that were directed at others but not mothers are also among those that were exclusively used in captivity, which also supports this conclusion. Given that Bornean orangutans are considered less gregarious and less socially tolerant than their North-Western Sumatran counterparts (van Schaik 1999; Fröhlich et al. 2020a), our findings also corroborate the view that widely different social affordances in terms of partner choice rather than just the physical properties of the research settings (flat ground and direct lines-of-sight, but also restricted space leading to high social density and thus a larger need for conflict regulation) shape immatures’ repertoire size and composition.

For both mother- and other-directed signal repertoires, we found no effect of orangutan species or research setting on the number of interaction outcomes (and thus social goals) immatures communicated towards. Although we cannot rule out an effect of the small sample size, this is an important finding given the differences in wild-captive contrasts on mother- versus other-directed signalling repertoires discussed above: similar contextual use of signals across settings would demonstrate that it is not just a larger variety of communicative contexts that captive orangutans are exposed to which drive the difference in repertoires between wild and captivity. In semi-solitary, fission–fusion species such as orangutans (*Pongo* spp.), interaction rates in contexts such as social play, grooming, conflict regulation and sex are presumed to be much higher in captive compared to naturalistic settings (e.g. Zucker et al. 1978; Maple 1980; Kopp and Liebal 2018; Fröhlich et al. 2021b). Thus, at first glance it would be plausible to assume that the higher sociability of zoo-housed orangutans alone, as well as the physical properties of the zoo setting (e.g. flat substrate, closer proximity and direct lines-of-sight) lead to the production of additional signals that are completely absent in the wild (Fröhlich et al. 2021a) and thus to differences in the communicative repertoires of individuals and groups. However, here, we provide some first evidence that it is the variety of interaction partners, and not just the mere exposure to flat ground or higher need or opportunity for communication afforded by captivity that foster more varied signal use: First, communication with mothers should have been equally affected by the wild-captive contrast, and second, there was no systematic wild-captive contrast in overall social goal variety. Therefore, based on these and similar recent findings in other primate taxa (Rebout et al. 2020; Grampp et al. 2023; Rincon et al. 2023), we argue that differentiated interactional opportunities may play a more pivotal role for the contrast in population- or individual-level repertoires than the mere number of conspecifics or social bonds with interaction partners. A such, these results add to recent work showing that the socio-ecological conditions linked to captivity lead to larger gestural and facial repertoires due to the production of additional, individually learned communicative utterances, which in our view must be linked at least to some degree of communicative innovativeness (see also Fröhlich et al. 2021a). We acknowledge that these conclusions are based on a rather small sample of immature orangutans, hence the inclusion of a larger variety of interaction dyads with larger samples will be needed in future studies to generalize this finding for whole populations or species.

Perhaps surprisingly, we found no strong effect of age on individual repertoire sizes in this sample of immatures. At first glance, this seems to contradict results from chimpanzees indicating that repertoire sizes increase throughout ontogeny, at least up to a certain age (e.g. Tomasello et al. 1997b; Fröhlich et al. 2017). The key explanation for the apparent lack of age effects in the orangutans of this study is probably that our sample did not include older and independent immatures, due to the lack of sufficiently large samples of mother-directed signals in these subjects.

As discussed before (Fröhlich et al. 2021a), a study on wild-captive contrasts comes with several caveats given that these two environments differ profoundly in conditions for behavioural observation (e.g. in the time that can be allocated to social interactions, as feeding competition in zoos is eliminated). In the present study, there were additional limitations regarding the analysis of individual signal repertoires instead of instances of signal use, which means that some important variables linked to *opportunity* (like the availability of social partners, see Fröhlich et al. 2020a) could not be assessed. When focusing on the size or composition of communicative repertoires (i.e. the variety of signal types used by individuals), we usually get only a single data point per individual. Only with extraordinary sampling efforts are we able to systematically split interaction partners into more fine-grained categories at least for a *subset of individuals*, like peer-directed or (non-mother)-adult-directed repertoires. This is very difficult to achieve in a model system such as the orangutan, which has an exceptionally slow life history characterised by long life spans and long inter-birth intervals (van Noordwijk et al. 2018). In our study, we attempted to split other-directed repertoires with regard to same-aged and older social partners and ended up comparing seven wild to eight captive peer-directed repertoires, and only four wild to twelve older-directed repertoires. Given this discrepancy in sample size, it is not surprising that the wild-captive contrast could only be replicated for peer-directed repertoires. In most zoos, immatures presumably interact more often with older non-mother individuals than in the wild. Thus, we cannot exclude the possibility that when the opportunity arises to interact with a rare adult partner in the wild, they might exhibit additional signal types as well. In the long run, the same kinds of interaction partners should be available for immatures in the wild, but it is much harder to sample the whole array due to very low frequency of availability. Here, the overall sample of mother-directed signals in the wild was about two or three times as large as the captive one, whereas the opposite was true for other-directed signals. Even so, this uneven dataset should not affect the validity of our conclusions, since we applied conservative rules of inclusion (i.e. signal used at least twice and more than thirty signal cases in each subject per partner category), and controlled for the number of contributed signal cases in all models.

Importantly, although an effect of sampling cannot be excluded and the opportunity to conduct ‘fair comparisons’ is limited, the wild-captive contrast in peer-directed repertoires indicates that it is not only the restricted availability of older partners in the wild driving the observed difference between wild and captive repertoires. Moreover, collinearity tests of predictor variables revealed that observation effort did not simply correlate with research setting, which would have suggested that wild-captive contrasts are merely caused by differences in sample sizes. Therefore, limitations imposed by studying communicative repertoires leave the central conclusion of this paper unaffected: that changes in signal repertoires crucially depend on the available interaction partners, not just the ecological setting great apes are exposed to. To our knowledge, teasing apart signal repertoires depending on interaction partners has not been attempted before, and here we provide the first tentative evidence for this, and call for replications.

Despite the increasing popularity of the social complexity hypothesis for the evolution of communication, the specific ways in which social factors impact communicative parameters are largely obscure. A myriad of possible associations between social (e.g. average group size, social organisation, social structure, mating system, care system) and communicative variables (e.g. number of signalling units, pattern complexity, combinations, gradation) have been identified in studies of birds, mammals, insects and amphibians (e.g. Blumstein and Armitage 1997; Dobson 2009; Ord and Garcia-Porta 2012), and researchers have expressed concerns that the complexity of these links may become oversimplified (Peckre et al. 2019). A fruitful research strategy may be to focus on suspected influential aspects, such as the degree of fission–fusion dynamics in relation to signalling complexity (Grampp et al. 2023), or the degree of social tolerance in relation to signal variability (Dobson 2012). Progress in this research area seems to depend on the clear specification of ‘what we are looking at’ (Peckre et al. 2019), as well as assessing communicative complexity not only based on the number of units but also on their respective connections (Freeberg et al. 2012; Pollard and Blumstein 2012). In previous work on the multimodality (Fröhlich et al. 2021b) and individuality of signalling (Fröhlich et al. 2022), we have already used research setting as a proxy for social complexity, contrasting wild (where individual-based fission–fusion dynamics are intact) with captive conditions (stable groups of up to nine individuals) in two species of orangutans reportedly diverging in sociability. In the present study, our proxies for communicative complexity were the signals and presumed social goals observed in interactions with two different recipient ‘classes’. The first, albeit preliminary, findings generated with this approach suggest that the regular availability of conspecifics other than the mother (‘social opportunities’) affects both repertoire size and composition, and as such adds one small piece to the puzzle on the relationship between social life and communicative behaviour. Importantly, testing associations between social and communicative variables may also inform current debates in the study of language origins and evolution (Freeberg et al. 2012; Peckre et al. 2019), given that the productivity feature of language (the ability to create and understand novel utterances with novel meanings; Hockett, 1960) reflects extreme communicative plasticity.

In conclusion, we explicitly and systematically examined communication systems of orangutans exposed to novel socio-ecological conditions relative to the wild baseline situation. Despite our limited sample of immature individuals, the present study shows that the previously identified wild-captive contrast in repertoire sizes can be largely explained by different social opportunities afforded by research settings, rather than being a by-product of differences in social goal variety and physical affordances. Again, this underscores the remarkable plasticity in the communicative behaviour of orangutans, as they seem to complement their species-typical repertoires with additional signals through individual learning (see also Fröhlich and van Schaik 2020; Fröhlich et al. 2021a). Moreover, these findings corroborate previous work in birds and mammals showing that not only social organisation (Blumstein and Armitage 1997; Krams et al. 2012) but also the characteristics of the targeted audience (Fröhlich et al. 2016; Crockford et al. 2017; Elie and Theunissen 2018; Ota et al. 2018) can have a profound impact on signalling behaviour. Future work including other social species beyond primates (and thus enabling larger sample sizes) will help to gain more insight into the specific ways social life shapes communicative abilities, and the extent to which nonhuman species are able to adjust their signalling in response to the current socioecological environment.

### Supplementary Information

Below is the link to the electronic supplementary material.

Supplementary file1 (DOCX 49 KB)

## Data Availability

Data and original code are publicly available on Zenodo with DOI 10.5281/zenodo.10413319. (https://zenodo.org/records/10413320).
