# Supplementary material for: Wild and captive immature orangutans differ in their non-vocal communication with others, but not with their mothers
Source: Behav Ecol Sociobiol. 2024 Jan 15;78(1):12. doi: 10.1007/s00265-023-03426-3 (PMC10789664; doi:10.1007/s00265-023-03426-3)
Supplement: Supplementary file 1 — Supplementary file1 (DOCX 49 KB) [file 265_2023_3426_MOESM1_ESM.docx]

**Supplementary Material**

**Wild and captive immature orang-utans differ in their non-vocal communication with others, but not with their mothers**

*Behavioral Ecology and Sociobiology*

Marlen Fröhlich^1,2^, Maria A. van Noordwijk^2,3^, Tatang Mitra Setia^4^, Carel P. van Schaik^2,3,5,6^, Ulrich Knief^7^

^1^ Paleoanthropology, Institute for Archaeological Sciences, Department of Geosciences, University of Tübingen, Tübingen, Germany

^2^ Department of Evolutionary Anthropology, University of Zurich, Zurich, Switzerland

^3^ Comparative Socioecology Research Group, Max Planck Institute of Animal Behavior, Konstanz, Germany

^4^ Fakultas Biologi, Universitas Nasional, 12520 Jakarta Selatan, Indonesia

^5^ Center for the Interdisciplinary Study of Language Evolution (ISLE), University of Zurich, Zurich, Switzerland

^6^ Department of Evolutionary Biology and Environmental Studies, University of Zurich, Zurich, Switzerland

^7^ Evolutionary Biology and Ecology, Faculty of Biology, University of Freiburg, Freiburg, Germany

Correspondence to: M. Fröhlich (marlen.froehlich@uni-tuebingen.de)**Supplementary Tables**

**Tab. S1** Information on study subjects and sample sizes (Bor = Bornean orang-utan, Sum = Sumatran orang-utan, N-mot = Number of observations for mother-directed signals; N-oth = Number of observations for other-directed signals).

| **No. subject** | **Setting** | **Species** | **Group** | **Age (yrs)** | **Sex** | **ID** | **N-mot** | **N-oth** |
| --- | --- | --- | --- | --- | --- | --- | --- | --- |
| 1 | captive | Bor | Apenheul | 2 | M | BAJ | 198 | 191 |
| 2 | captive | Bor | Cologne | 5 | F | CIT | 201 | 776 |
| 3 | captive | Bor | Cologne | 4 | F | CIR | 109 | 624 |
| 4 | captive | Bor | Munster | 4 | F | NIA | 0 | 445 |
| 5 | captive | Bor | Munster | 3 | M | MIY | 106 | 682 |
| 6 | captive | Sum | Munich | 1 | F | RON | 106 | 85 |
| 7 | captive | Sum | Munich | 1 | M | QUE | 38 | 246 |
| 8 | captive | Sum | Munich | 1 | M | QUI | 24 | 77 |
| 9 | captive | Sum | Zurich | 6 | F | MIM | 32 | 168 |
| 10 | captive | Sum | Zurich | 6 | M | MAL | 33 | 302 |
| 11 | captive | Sum | Zurich | 3 | F | PAN | 35 | 132 |
| 12 | captive | Sum | Zurich | 1 | F | RIA | 43 | 54 |
| 13 | wild | Bor | Tuanan | 6 | M | TUK | 47 | 10 |
| 14 | wild | Bor | Tuanan | 4 | F | JAN | 147 | 78 |
| 15 | wild | Bor | Tuanan | 3 | F | MOB | 190 | 183 |
| 16 | wild | Bor | Tuanan | 3 | M | CAK | 124 | 0 |
| 17 | wild | Bor | Tuanan | 2 | M | DAR | 325 | 5 |
| 18 | wild | Bor | Tuanan | 1 | M | KEC | 54 | 0 |
| 19 | wild | Bor | Tuanan | 3 | M | KET | 384 | 77 |
| 20 | wild | Bor | Tuanan | 2 | M | MER | 313 | 105 |
| 21 | wild | Sum | Suaq | 6 | F | CIN | 108 | 96 |
| 22 | wild | Sum | Suaq | 5 | M | FRA | 142 | 213 |
| 23 | wild | Sum | Suaq | 7 | M | LOI | 69 | 71 |
| 24 | wild | Sum | Suaq | 3 | F | EDE | 297 | 72 |
| 25 | wild | Sum | Suaq | 2 | M | LUT | 210 | 79 |
| 26 | wild | Sum | Suaq | 6 | M | PEP | 33 | 0 |
| 27 | wild | Sum | Suaq | 3 | M | TOR | 78 | 2 |

*Note: NIA includes no mother-directed signals since her mother died right before the start of data collection.*

**Tab. S2** Orang-utan immatures’ non-vocal signal repertoires based on Fröhlich et al. (2021), broken down by interaction partner, species and research setting. Signal types never directed at mothers are depicted in italics.

|  | **Mother-directed** | | | | **Other-directed** | | | |
| --- | --- | --- | --- | --- | --- | --- | --- | --- |
|  | Bornean | | Sumatran | | Bornean | | Sumatran | |
| **Non-vocal signal** | Captive | Wild | Captive | Wild | Captive | Wild | Captive | Wild |
| Beg hand-hand | 10 | 141 | 20 | 64 | 32 |  | 23 | 8 |
| Beg hand-mouth | 13 | 80 | 14 | 64 | 13 |  | 25 | 3 |
| Beg mouth-hand | 5 | 67 | 39 | 19 | 21 |  | 30 | 5 |
| Beg mouth-mouth | 12 | 35 | 43 | 14 | 29 |  | 65 | 8 |
| Bite | 24 | 193 | 8 | 3 | 71 | 30 | 27 | 2 |
| Bite attempt | 27 | 52 | 6 | 6 | 45 | 17 | 26 | 8 |
| Dangle | 13 | 25 | 3 | 29 | 256 | 11 | 37 | 29 |
| Embrace | 2 | 7 | 3 | 15 | 3 |  | 5 | 1 |
| *Flap lip* |  |  |  |  |  |  | 1 | 1 |
| Fling | 5 | 2 | 1 | 9 | 12 | 2 | 25 | 23 |
| Grab/hold | 161 | 519 | 50 | 200 | 445 | 82 | 75 | 75 |
| Hand on | 50 | 122 | 7 | 113 | 76 | 11 | 9 | 20 |
| Head-butt |  |  | 4 | 3 | 1 |  | 19 | 2 |
| *Head-stand* |  |  |  |  | 8 |  |  | 1 |
| Hit | 7 |  | 7 | 1 | 52 |  | 57 | 1 |
| *Hit ground/object* |  |  |  |  | 2 |  |  |  |
| Kiss | 4 | 14 | 5 |  | 7 | 1 | 32 |  |
| Look at | 15 | 12 | 3 | 102 | 148 | 2 | 32 | 67 |
| Look back at | 4 |  |  |  | 8 |  | 11 | 2 |
| Loud scratch |  |  |  | 5 |  |  |  | 1 |
| Peer | 18 | 38 | 28 | 63 | 290 | 1 | 134 | 16 |
| Play face | 3 | 9 | 7 | 2 | 61 | 1 | 34 | 17 |
| Poke | 5 | 24 | 4 |  | 110 |  | 44 | 3 |
| Pout face |  | 7 | 2 |  | 1 |  | 4 |  |
| Present body part | 8 | 20 | 8 | 11 | 10 | 23 | 7 | 10 |
| Present object | 1 | 1 |  | 3 | 17 |  | 6 |  |
| Pull | 43 | 10 | 12 | 55 | 67 | 5 | 99 | 37 |
| Push | 25 | 6 | 1 | 10 | 31 | 1 | 17 | 25 |
| Raise limb | 26 |  | 1 | 5 | 122 |  | 6 | 3 |
| Reach | 11 | 9 | 4 | 28 | 142 | 4 | 46 | 55 |
| Rise up | 6 |  |  |  | 48 |  | 1 |  |
| Roll on back | 16 |  |  | 1 | 77 |  | 1 |  |
| Rub on rec |  |  | 4 | 8 |  |  | 49 | 16 |
| *Shake object* |  |  |  |  |  |  |  | 2 |
| Somersault | 5 |  | 1 |  | 50 |  | 3 |  |
| *Spin* |  |  |  |  | 7 |  |  |  |
| *Spit* |  |  |  |  | 1 |  |  |  |
| Stroke | 2 | 2 |  | 4 | 4 |  |  | 1 |
| Throw object | 1 | 1 |  |  | 41 |  | 7 |  |
| Throw self | 19 |  | 13 |  | 107 |  | 36 | 1 |
| Touch | 73 | 188 | 13 | 100 | 303 | 266 | 71 | 90 |
| Total | 614 | 1584 | 311 | 937 | 2718 | 458 | 1064 | 533 |

**Tab. S3** Effects of age, research setting, orang-utan species and control predictors on individual repertoire sizes directed at conspecifics other than the mother, derived using GLMMs with a Poisson error structure. Significant effects (P < 0.05) are depicted in italics. NA: Values have no meaningful interpretation.

|  | Estimate | S.E. | *χ^2^_1_* | *P* |
| --- | --- | --- | --- | --- |
| Intercept | 2.613 | 0.162 | NA | NA |
| Age | 0.021 | 0.083 | 0.062 | 0.803 |
| Age^2^ | -0.084 | 0.098 | 0.746 | 0.388 |
| Sex [male] | 0.134 | 0.128 | 1.094 | 0.296 |
| *Setting [wild]* | *-0.420* | *0.191* | *4.837* | *0.028* |
| *Species [Sum]* | *0.518* | *0.171* | *8.124* | *0.004* |
| *No. signal cases* | *0.349* | *0.108* | *7.411* | *0.006* |

**Tab. S4** Overview of coders, coded study groups and inter-coder reliability.

| Observer | Study group | κ  presumed goal | κ  signal type | κ response |
| --- | --- | --- | --- | --- |
| Lauren Momon | Suaq | 0.82 | 0.76 | 0.61 |
| Natasha Bartolotta | Suaq | 0.87 | 0.89 | 0.76 |
| Marvin Jaffrezic | Tuanan | 0.8 | 0.89 | 0.65 |
| Caroline Fryns | Zurich | 0.9 | 0.87 | 0.77 |
| Santhosh Totagera | Munich | 0.82 | 0.96 | 0.78 |
| Elisa Dore | Apenheul, Cologne, Munster | 0.93 | 0.83 | 0.78 |
| **Mean** |  | **0.86** | **0.87** | **0.73** |

**References**

Fröhlich, M., Bartolotta, N., Fryns, C., Wagner, C., Momon, L., Jaffrezic, M., Mitra Setia, T., Schuppli, C., Noordwijk, M. A., & van Schaik Carel, P. (2021). Orangutans have larger gestural repertoires in captivity than in the wild – a case of weak innovation? *iScience*, *24*(11), 103304. <https://doi.org/10.1016/j.isci.2021.103304>
